# Accelerometry and the Capacity–Performance Gap: Case Series Report in Upper-Extremity Motor Impairment Assessment Post-Stroke

**DOI:** 10.3390/bioengineering12060615

**Published:** 2025-06-04

**Authors:** Estevan M. Nieto, Edaena Lujan, Crystal A. Mendoza, Yazbel Arriaga, Cecilia Fierro, Tan Tran, Lin-Ching Chang, Alvaro N. Gurovich, Peter S. Lum, Shashwati Geed

**Affiliations:** 1 Department of Rehabilitation Sciences, The University of Texas at El Paso, El Paso, TX 79968, USA; emnieto3@miners.utep.edu (E.M.N.); elujan11@miners.utep.edu (E.L.); camendoza15@miners.utep.edu (C.A.M.); yarriaga2@miners.utep.edu (Y.A.); 2 Department of Occupational Therapy, The University of Texas at El Paso, El Paso, TX 79902, USA; cfierro6@utep.edu; 3 Department of Computer Science, The Catholic University of America, Washington, DC 20064, USA; 38tran@cua.edu (T.T.); changl@cua.edu (L.-C.C.); 4 Department of Informatics, New Jersey Institute of Technology, Newark, NJ 07102, USA; 5 Department of Physical Therapy and Movement Sciences, The University of Texas at El Paso, El Paso, TX 79968, USA; agurovich@utep.edu; 6 Department of Biomedical Engineering, The Catholic University of America, Washington, DC 20064, USA; lum@cua.edu

**Keywords:** stroke, neurorehabilitation, accelerometry, motor recovery, stroke rehabilitation trials, motor outcomes

## Abstract

This case series investigates whether traditional machine learning (ML) and convolutional neural network (CNN) models trained on wrist-worn accelerometry data collected in a laboratory setting can accurately predict real-world functional hand use in individuals with chronic stroke. Participants (N = 4) with neuroimaging-confirmed chronic stroke completed matched activity scripts—comprising instrumental and basic activities of daily living—in-lab and at-home. Participants wore ActiGraph CenterPoint Insight watches on the impaired and unimpaired wrists; concurrent video recordings were collected in both environments. Frame-by-frame annotations of the video, guided by the FAABOS scale (functional, non-functional, unknown), served as the ground truth. The results revealed a consistent capacity–performance gap: participants used their impaired hand more in-lab than at-home, with the largest discrepancies in patients with moderate to severe impairment. Random forest ML models trained on in-lab accelerometry accurately classified at-home hand use, with the highest performance in mildly and severely impaired limbs (accuracy = 0.80–0.90) and relatively lower performance (accuracy = 0.62) in moderately impaired limbs. CNN models showed comparable accuracy to random forest classifiers. These pilot findings demonstrate the feasibility of using lab-trained ML models to monitor real-world hand use and identify emerging patterns of learned non-use—enabling timely, targeted interventions to promote recovery in outpatient stroke rehabilitation.

## 1. Introduction

Randomized clinical trials are the gold standard used to establish effective interventions for post-stroke motor restoration. Robust outcome measures are the backbone of any clinical trial. Conventionally, stroke rehabilitation trials have relied on clinical scales like the Action Research Arm Test (ARAT) [1] or Upper-Extremity Fugl–Meyer (UEFM) test [2]. Although the clinical scales are psychometrically robust [3,4,5,6], they suffer from ceiling and floor effects when measuring motor recovery over time in the context of clinical trials [7] that enroll patients across a range of upper-extremity (UE) motor impairments. Additionally, patients with similar clinical scores on the ARAT or UEFM express a wide range of proficiency in everyday activities [8].

The in-clinic measurement of patients’ motor abilities, called *capacity*, is often different from their actual *performance* in activities and instrumental activities of daily living (ADL/IADLs) in their own homes and community [9,10]. While capacity is useful for clinicians evaluating a patients’ recovery, in-clinic recovery does not translate well to understanding recovery that patients care about—their ability to perform their preferred ADL/IADLs in their own homes and communities. This gap between what patients can accomplish in the clinic (capacity) and what they actually do with their impaired limb in their own homes (performance) is well recognized in neurorehabilitation [9,11,12,13]. In this scenario, clinical trials measuring an intervention’s efficacy or effectiveness must rely on both clinical capacity as well as the patients’ performance in their preferred ADL/IADLs in their own homes. However, major UE stroke rehabilitation trials to date have utilized capacity-centric measures as their primary outcome [14,15,16,17,18], highlighting the need for a clinically and ecologically valid outcome of motor function post-stroke that captures motor recovery in patients’ own homes.

Wrist-worn accelerometers are compact, smartwatch-sized devices that measure acceleration along the x, y, and z axes of the wrist. Accelerometers have been used to quantify upper-extremity movements in individuals with stroke [8,19,20,21,22], cerebral palsy [23,24,25], and orthopedic conditions [26]. When worn on both the impaired and unimpaired wrists, these accelerometers continuously track upper-extremity movement, allowing for the calculation of a use ratio—the proportion of impaired to unimpaired hand movement—which serves as a proxy for functional recovery [27]. However, not all detected movement reflects meaningful motor recovery. To address this limitation, we developed and clinically validated traditional machine learning and convolutional neural network (CNN)-based approaches to classify accelerometry data from impaired/unimpaired arms into functional and non-functional use [28]. We have previously shown that the amount of functional hand use as an outcome is superior to conventional upper-extremity accelerometry *counts* [28], and that it is both clinically reliable and valid in individuals with chronic stroke [8].

In the present case series, we test whether traditional machine learning and CNN models trained on accelerometry data recorded in a clinical lab setting can accurately predict functional hand use in patients’ home environments. A crucial limitation of real-world performance outcomes is the logistical and privacy challenges in recording data wherever the patients may be situated at a given point in time. For stroke rehabilitation trials to implement outcomes that capture real-world performance, it is crucial that the measure accurately captures meaningful arm function in the patients’ everyday life but does not encroach on patients’ or caregivers’ privacy and is logistically feasible. By testing if in-lab accelerometry accurately predicts at-home data in a small cohort of mildly, moderately, and severely impaired chronic stroke patients, this study takes a crucial first step towards establishing its ecological validity as a measure of functional hand use in real-world settings.

## 2. Materials and Methods

### 2.1. Participants

Individuals were recruited from the University Medical Center of the El Paso outpatient service using recruitment flyers. The inclusion criteria were (1) neuroimaging-confirmed ischemic or hemorrhagic stroke, (2) an age of more than 18 years at the time of providing informed consent, (3) no known orthopedic or neuromuscular injuries that interfered with the completion of study procedures, and (4) a Mini-Mental status examination score > 24. Individuals were excluded if (1) they exhibited neglect as determined by an asymmetry > 3 errors on Mesulam’s symbol cancelation test, (2) had had a prior stroke with residual motor impairment, or (3) received botulinum toxin during the study. The study was conducted in accordance with the Declaration of Helsinki and approved by the Institutional Review Board of the University of Texas at El Paso (protocol number: 2127649; approval date: 2 April 2024). Written informed consent was obtained from all individuals tested in the study.

### 2.2. Apparatus and Measures

**Figure 1:**
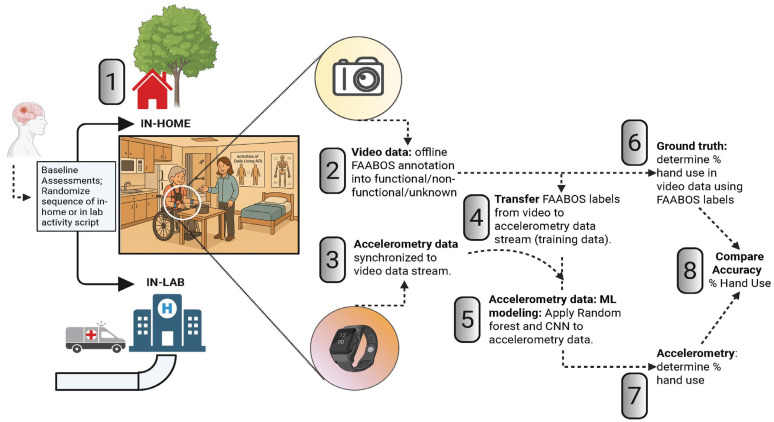
Overview of study data collection and analysis. 1. Participants completed two data collection sessions approximately 5–6 days apart: one in-lab and one at-home. In Session 1 (in-lab), upper-extremity (UE) motor function was assessed using clinical tests (Action Research Arm Test [ARAT], Upper Extremity Fugl–Meyer [UEFM] test, and Edinburgh Handedness Inventory). Participants then completed an Activity Script simulating daily living tasks (e.g., meal prep, laundry, shopping, typing) while wearing wrist-mounted accelerometers and a chest-mounted video camera. Session 2 replicated these tasks in the participants’ own homes using their own tools and equipment. 2. Video data were annotated offline by trained raters using a modified Functional Arm Activity Behavioral Observation System (FAABOS) and classified as functional, non-functional, or unknown use. These labels served as the ground truth. 3. Accelerometry data were synchronized to the videos using a standardized movement protocol, segmented into 2 s blocks, and labeled based on the majority ground truth classification. Machine learning models (random forest [RF] and convolutional neural network [CNN]) were trained using in-lab data, validated via 5-fold cross-validation and tested both within-session and on the at-home session data to assess generalizability (4–5). Model accuracy—the proportion of correctly classified time blocks—was compared across sessions and algorithms (6–8).

Figure 1 describes the flow of study data collection and analysis.

#### 2.2.1. Clinical Testing

Data were collected over two sessions approximately 5–6 days apart. The first session was completed in-lab, and the second session was completed at the patient’s home. During the first session, upper-extremity motor function was evaluated using the Action Research Arm Test (ARAT, a clinical test of UE motor performance) [1,3,29] and the Upper Extremity Fugl–Meyer test (UEFM, a clinical test of motor impairment) [2]. The Edinburgh Handedness Inventory [30,31] was used to determine the concordance between handedness and the side of the impairment—that is, whether the dominant arm was affected. The order of testing (at-home versus in-lab) was randomly selected.

#### 2.2.2. Activity Script:

Participants completed an activity script twice, first in-lab (session #1) and a second time, 5–6 days later, at their homes (session #2). The activity script is described in detail in our prior reports [28]. Briefly, the activity script is a set of ADL/IADLs used to simulate functional UE use in the community. The in-lab activity script was completed in a simulated apartment at the UTEP Department of Occupational Therapy. The simulated apartment houses a fully functional “living space” including a kitchen, bedroom, laundry, a store front for shopping activities, and a dining area with appropriate furniture for meals. Individuals were instructed to perform the following IADLs during the activity scripts completed in-lab and at-home: (1) a laundry task, (2) linen management, (3) grocery shopping, (4) a kitchen meal preparation task, (5) financial management, (6) medication management, and a (7) typing task. Participants were instructed to perform the activities as they would naturally, with no specific instructions as to which arm to use and how to complete the task. Participants were instructed to, e.g., “prepare a sandwich and pour a glass of water/juice to go with the sandwich for lunch”. There was no set time limit for participants to complete the activity. Between the activity script tasks, participants sat at the dining table in the simulated apartment while the experimenters engaged them in conversation. Participants also walked around the facility to collect non-functional UE movement data. Similar activities were completed during the at-home session (session #2) with similar instructions. Participants completed the at-home activity script using their own equipment and tools.

#### 2.2.3. Accelerometry and Video Data Recording

Accelerometry and video recording during activity script performance: Participants wore wireless accelerometers (ActiGraph CenterPoint Insight Watch, Pensacola, FL, USA), similar to a smartwatch, on both wrists while performing the activity script tasks in-lab and at-home. The accelerometers are sensitive to movement in three axes, with raw acceleration sampled at 32 Hz and stored for offline processing. Raw data from the watches is transmitted to the CenterPoint cloud upon completing the activity script for both in-lab and at-home data collections. Simultaneously with accelerometry, participants video-recorded the activity of their own arms using a Kodak PixPro SP360 4K camera (JK Imaging Ltd., Gardena, CA, USA) mounted securely to a chest harness worn by the participants. Video data were recorded and stored at 30 Hz for offline processing. To enable the offline synchronization of video and accelerometry data streams, the experimenter held both accelerometers and moved them up and down (vertically) 5 times in view of the video before placing the watches on the patients’ hands at the start of the activity script and after removing the watches from the patients’ hands at the end of data collection.

### 2.3. Data Processing

#### 2.3.1. Video Annotation

A recorded video of the activity script was annotated by trained research assistants (EMN, EL, CAM, YA) using a standardized coding scheme based on the Functional Arm Activity Behavioral Observation System (FAABOS) [32], as described in our prior reports [8,28]. Briefly, the activity script video was watched offline by two trained research assistants. Each frame was labeled according to the five FAABOS categories, which were subsequently collapsed into three categories (functional, non-functional, or unknown hand use). Functional activities included any reaching to grasp or prehension, such as pushing open a door, opening or closing jars, etc. (purposeful upper-extremity activities contributing to the desired task). Non-functional periods included rest and arm movements associated with gait, gestures, and purposeless movements not directly related to the desired task. Although these “non-functional” tasks may reflect meaningful activity, they were considered non-functional in this context as they preclude prehension, which is the target of interventions in conventional upper-extremity stroke rehabilitation trials. The right and left upper extremities were coded separately frame-by-frame by two annotators independently. Any discrepancies were resolved by a third trained annotator (majority vote). The final coding values allowed the % functional use ratio to be calculated, defined as the % of functional use in the impaired limb normalized to the less impaired UE. These video-annotated labels are referred to as ground truth use ratios.

#### 2.3.2. Application of Machine Learning Algorithms to Accelerometer Data

**Figure 2:**
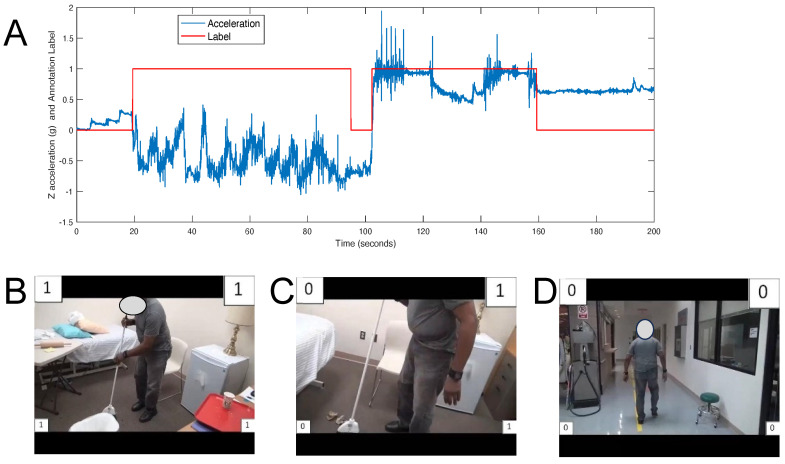
Superposition of accelerometry and video-labeled FAABOS categories. (**A**) Sample z-acceleration signal with overlayed annotation label (0 = non-functional, 1 = functional) for the right upper extremity in a representative participant. Notice the transitions in FAABOS labels corresponding to clear changes in the acceleration signal. (**B**–**D**) Single video frames of a participant performing activity script tasks during the FAABOS states shown in (**A**). The left-arm FAABOS labels are on the left side of the frame; the right-arm labels are on the right side of the frame. Top labels are ML model predictions; bottom labels are human annotations (trained research assistants).

We developed machine learning models with the accelerometry data. To synchronize video and accelerometry data streams, the experimenter held both accelerometers and moved them up and down in the vertical dimension 5 times in view of the video. The maximum and minimum acceleration values corresponding to reversals in movement direction were both easily identified in the video and accelerometry data streams during offline processing. This synchronization movement was performed before placing the accelerometers on the subject and again after they were removed. Ground truth annotation labels were moved from video frames to the synchronized acceleration data points. Figure 2 shows the human-annotated FAABOS labels superimposed on the z-axis accelerometry signal after synchronization.

No filtering was performed on the raw accelerometry data. The acceleration data was then partitioned into 2 s blocks, and 17 features were calculated for each block. These were based on prior work [8] and included the mean, variance, max, and min of each acceleration axis. Similar features were calculated from the Euclidean norm of the 3 acceleration axes. System entropy was also calculated as an additional feature. If more than 90% of labels in a 2 s block were in the same class, that label was applied to the block. The remaining blocks were excluded from the analysis. In our prior work, the random forest (RF) classifier was found to be the best overall algorithm for within-subject modeling [8]; we therefore applied the random forest approach to these data. To compare performance and address potential data noise, we also developed a 4-layer convolutional neural network (CNN). Due to the relatively small dataset size, we incorporated Batch Normalization to stabilize training and used L2 regularization and dropout to prevent overfitting. Within-subject models were applied to each data collection session. Five-fold cross validation was used to train and validate the model, which tested the accuracy of the model when applying it to new data not in the training set but from the same subject and under the same experimental conditions (i.e., collected in the lab). We also trained a model on the in-lab data and tested it on data from the same subject in the home setting. This tests if the in-lab model is generalizable to home data collected from the same subject. We calculated several performance metrics from each test. Accuracy is the ratio of correct classifications to total cases.

#### 2.3.3. Statistical Analysis

This is a case series from a cohort of N = 4 patients with chronic stroke. Descriptive statistics and raw data are presented in this preliminary report, comparing the accuracy of in-lab to at-home data on functional hand use.

## 3. Results

Demographic, clinical, and UE activity characteristics for the patients are shown in Table 1. We enrolled four patients (one female, mean age ± SD = 65.25 ± 13.2 years) with ischemic stroke (mean time since stroke = 49.5 ± 53 months, range = 7.5 months to 10.5 years). Participants spanned a range of impairment levels. The most severely impaired patient had an impaired-side ARAT score = 9/56 and an impaired-side UEFM score = 22/66. The most mildly impaired patient showed an ARAT score on the impaired side = 56/56 and UEFM = 60/66. All patients experienced impairment in their dominant arm (concordant).

### 3.1. Video (Ground Truth) Data Shows a Capacity–Performance Gap In-Lab and At-Home

**Figure 3:**
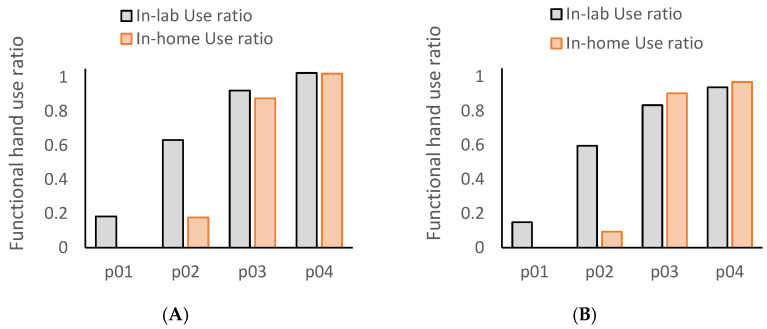
Video- and accelerometry-based functional hand use ratios for in-lab and at-home activity scripts. (**A**) Video-based ground truth use ratios. (**B**) Functional hand use ratios calculated by the random forest model applied to accelerometry data. Functional hand use is calculated as the % of functional use in the impaired limb normalized to the less-impaired UE. The at-home use ratio could not be calculated for p01 because their at-home impaired hand use in functional tasks was zero.

Table 2 shows the amount of functional hand use calculated from the video data streams (ground truth). The video ground truth showed that the amount of functional hand use was lower at-home compared to in-lab in each of the four patients. The capacity–performance difference showed a gradient such that the moderately impaired patients experienced the greatest % difference in the amount of hand use in-lab compared to at-home. P02, with a moderately impaired UE (ARAT = 40/56), had an in-lab use ratio = 0.63, but the at-home use ratio dropped to 0.17, a 35% reduction. P01 (ARAT = 9/56) had an in-lab use ratio = 0.18 but showed 0 use of the impaired hand at-home, an 18% reduction. Similarly, p03 and p04 both experienced mild motor impairments (ARAT = 56 and 50/56, respectively); their in-lab use ratios of 0.92 and 1.03, respectively, dropped to 0.87 and 1.02, respectively, for at-home use. Figure 3A shows the % hand use ratios in-lab and at-home based on video annotations of data (ground truth).

### 3.2. Accelerometry-Based Amount of Functional Hand Use In-Lab and At-Home

Table 3 shows the accelerometry predictions of in-lab use % in the paretic limb (predicted from the model trained using in-lab accelerometry data) and at-home use % (predicted from the model using at-home accelerometry data). Accuracies were computed against the corresponding video (ground truth) data. Both the RF and CNN classifiers showed high accuracies for in-lab and at-home data when using models trained on their own kind (in-lab to in-lab; at-home to at-home). The RF and CNN models performed similarly for in-lab data across all subjects. The CNN tended to slightly outperform RF at-home for subjects with moderate use (p02, p03), but it underperformed for the subject with high use (p04).

### 3.3. Prediction of At-Home Functional Hand Use from Machine Learning Models Trained on In-Lab Data

**Figure 4:**
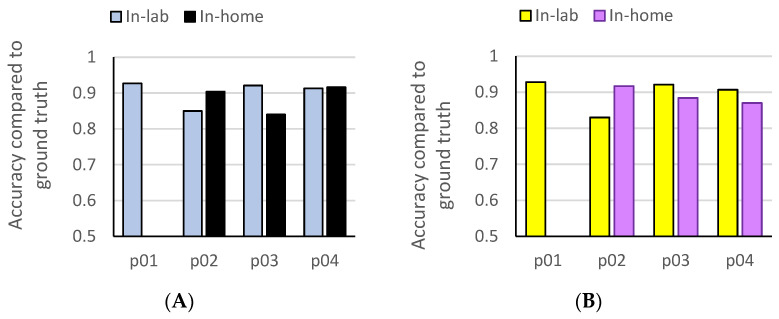
Accuracy of random forest and convolutional neural network classification of in-lab and at-home accelerometry compared to video-based ground truth. (**A**) Accuracy of random forest models. (**B**) Accuracy of convolutional neural network models.

Table 4 shows the classification accuracy and the functional hand use % predicted for the at-home activity script using the same patients’ in-lab machine learning models. Absolute errors of predicted functional use were also computed to compare the RF and CNN models. The RF classifiers trained using in-lab data showed relatively high accuracies in predicting at-home functional use %, especially in the very severely impaired (p01, accuracy = 0.98) and least severely impaired (p03, accuracy = 0.80; p04, accuracy = 0.87) patients. The at-home prediction accuracy was relatively lower for the moderately impaired patients (p02, 0.62) compared to the predictions for the more and less severely impaired patients (p01, p03, p04). The CNN model performed slightly better than RF in classification accuracy, especially for high-use participants (p03, p04). The consistency between models was high for participants with moderate to high use (p02, p03, p04), but the CNN performed less well in the subject with low use (p01). Figure 4 shows the accuracy of random forest and convolutional neural network classification of in-lab and at-home accelerometry compared to video-based ground truth for each of the study participants.

## 4. Discussion

Both the RF and CNN models classifying functional hand use that were trained using in-lab accelerometry data showed relatively high accuracies in predicting at-home hand use, albeit in a small cohort of stroke patients. The results also highlight the capacity–performance gap in motor function recovery post-stroke. Patients tended to use their impaired upper extremity more in-lab than in their own homes, as evidenced through the amount of functional hand use measured on the video (ground truth)-labeled activity scripts. The % difference between in-lab and at-home use was greatest for the moderately and severely impaired patients; however, the gap persisted even in the least impaired patient’s hand use despite them having a nearly perfect hand use ratio in-lab. Although these results come from a small cohort of stroke patients, they span the most severe, moderate, and least severe ends of the UE motor function impairment spectrum.4.1. Implications for Measuring Motor Recovery in the Patients’ Own Homes

The extent to which patients use their impaired upper extremity in meaningful daily activities is a crucial measure of neurorehabilitation efficacy. Our findings advance accelerometry-based assessments by demonstrating that machine learning models trained on in-lab data can accurately predict functional hand use in patients’ homes, albeit in a small cohort. This patient-centered outcome represents a significant improvement over existing methods for measuring at-home motor performance. Currently, few stroke rehabilitation outcomes objectively quantify motor function outside the clinic. While self-reported measures such as the Motor Activity Log [32] and the Stroke Impact Scale [33,34] are used, they are susceptible to recall biases and may not be ideal for assessing function in the acute and subacute post-stroke phases—critical periods targeted by recent large-scale randomized controlled trials in upper-extremity stroke rehabilitation [14,15,16,35].

Furthermore, assessing motor function at patients’ homes in stroke rehabilitation trials presents significant privacy and logistical challenges. At-home testing is often impractical, making it highly advantageous to use machine learning models trained on accelerometry data from a brief, 20 min scripted activity in a lab or clinical setting to accurately predict patients’ hand use during ADLs and IADLs in their home environments. An objective at-home outcome on the amount of functional hand use reduces the testing burden for patients and trialists, minimizing the odds of missing data in longitudinal stroke recovery trials, which typically measure recovery up to 12 months post-stroke [14,15,16,35]. A quantitative outcome of at-home performance that is valid both clinically and in the home environment is essential for clinical trials focused on effective rehabilitation interventions after stroke.

The RF model’s accuracy ranged from 0.8 to 0.9 for the three severe or mildly impaired patients. Accuracy was relatively lower for the moderately impaired patient (p02, 0.62). Given the small sample size tested in this case series, it is likely that a larger cohort will introduce greater variability in prediction accuracy across all impairment levels. The high prediction accuracy in severely impaired patients may be attributed to the limited repertoire of motor strategies available for severely impaired limbs. With an ARAT score of 9/56, this severely impaired patient exhibited severely constrained use of their upper extremity, whether in-lab or at-home, leading to high prediction accuracy for at-home data using models trained on in-lab data. Similarly, the mildly impaired patients (p03, ARAT = 50; p04, ARAT = 56) with high use ratios experienced minimal constraints on their use of the upper extremity, whether in-lab or in their homes, resulting in higher prediction accuracy for at-home hand use based on in-lab data.

In contrast, the lower prediction accuracy for moderately impaired arms may be due to *learned non-use*, a real capacity–performance gap where patients use the impaired limb when explicitly tested in-lab but revert to non-use in familiar home environments. This hypothesis is supported by video-based ground truth data, which shows a substantial decline in the patient’s upper-extremity use ratio from 0.63 in-lab to 0.17 at-home. These differences between in-lab and at-home use might explain why the model based on lab data had the lowest accuracy for predicting at-home data for the individual with moderate impairment. A larger sample with a broader range of stroke impairments will help clarify the underlying mechanisms driving this capacity–performance gap. In fact, the data shows that the motor capacity of patients improves over several months post-stroke, but performance in ADL/IADLs stalls [9]. Access to accurate, real-world performance metrics could enable clinicians to identify and address capacity–performance mismatches more strategically during rehabilitation.

To test whether moderately impaired patients consistently demonstrate a capacity–performance divergence, future studies need to incorporate longitudinal measures of upper-limb use through machine learning-based accelerometry in both clinical and home settings at strategic intervals of time post-stroke (e.g., 1, 2, 3, 4, 6, and 12 months). A longitudinal design would allow for detailed modeling of how performance evolves in relation to motor capacity, helping to identify critical periods when learned non-use is most likely to emerge. Detecting these periods could inform the timing of targeted interventions and assess whether early, feedback-based strategies can mitigate the onset or severity of learned non-use and positively influence recovery trajectories.

The CNN model demonstrated comparable or slightly higher accuracy in most cases and showed an advantage in detecting functional use in participants with mild to moderate impairment (p03, p04). While the RF model predicted a very low functional use ratio (2%) for the severely impaired arm (p01), the CNN model predicted a substantially higher use ratio (31%), suggesting that the CNN may overestimate functional use by misclassifying subtle or ambiguous movements in cases of low activity. For subject p02, both RF and CNN showed limited generalization, supporting the hypothesis of a true capacity–performance gap in the moderately impaired patients. Overall, the CNN offers a promising alternative to RF, eliminating the need for manual feature extraction; however, further validation on larger datasets is necessary to assess its generalizability.

In summary, an RF- and CNN-based accelerometry approach offers a powerful tool for objectively quantifying patient-centered outcomes for stroke rehabilitation trials and clinical care in neurorehabilitation. Beyond measurement, it holds the potential to track the progression of learned non-use and capacity–performance gaps throughout post-stroke motor recovery. By enabling the timely detection of declines in upper-extremity use within patients’ real-world home environments, this technology could help clinicians intervene earlier—whether through adaptive cues or at-home modifications—to maximize recovery and prevent the re-emergence of learned non-use. In the future, integrating this approach into rehabilitation protocols (e.g., through remote monitoring platforms or electronic health records) could personalize treatment strategies, ensuring sustained and meaningful improvements in functional hand use.

### Limitations

The small sample size in this study limits the generalizability of the findings. Although the cohort included individuals with mild, moderate, and severe upper-extremity impairments, the heterogeneity of stroke recovery and functional environments warrants caution in interpreting the results. Future longitudinal studies with larger and more diverse samples are needed to validate these findings and further refine predictive models of real-world hand use post-stroke.

## 5. Conclusions

This traditional machine learning- and CNN-based accelerometry approach is a valuable tool to not only quantify patient-centric outcomes objectively for clinical trials and clinical care but also to investigate the timeline and patterns of learned non-use or capacity–performance gaps in post-stroke motor recovery. The timely identification of declines in the use of the impaired upper extremity in the patients’ environments can alert clinicians of the need to introduce interventions, cues, and at-home adaptations to ensure maximal recovery and that patients do not revert to learned non-use.

## Figures and Tables

**Table 1:** Demographic and clinical characteristics of patients participating in the study, with age at the time of consent. Race AA = African American. Concordant stroke = dominant upper extremity (determined using the Edinburgh Handedness Inventory) is more impaired. ARAT: Action Research Arm Test. UEFM: upper-extremity Fugl–Meyer. PID: Participant ID.

PID	p01	p02	p03	p04
Age (years)	76	68	46	71
Sex	Female	Male	Male	Male
Race	AA	White	White	White
Ethnicity	Hispanic	Hispanic	Hispanic	Hispanic
Stroke type	Ischemic	Ischemic	Ischemic	Ischemic
Months post-stroke	126.9	7.5	26.2	37.5
Affected arm	Dominant	Dominant	Dominant	Dominant
Concordance	Concordant	Concordant	Concordant	Concordant
NIHSS motor arm (Impaired)	3	2	1	1
NIHSS motor arm (Unimpaired)	0	0	0	0
ARAT (Impaired)	9	40	56	50
ARAT (Unimpaired)	56	57	57	57
UEFM (Impaired)	22	48	60	58
UEFM (Unimpaired)	66	66	66	66

**Table 2:** Functional use ratios in-lab and at-home from video (ground truth) data.

PID	Functional Use from Video (Ground Truth)					
	In-Lab			At-Home		
	Impaired	Unimpaired	Use Ratio	Impaired	Unimpaired	Use Ratio
p01	0.173	0.943	0.183	0	0.864	0
p02	0.611	0.967	0.631	0.149	0.838	0.177
p03	0.765	0.830	0.922	0.820	0.936	0.876
p04	0.885	0.863	1.025	0.922	0.903	1.021

**Table 3:** Functional use from accelerometry in-lab and at-home data.

In-Lab Data						At-Home Data				
		Use %		Accuracy			Use %		Accuracy	
PID	# of Samples	RF	CNN	RF	CNN	# of Samples	RF	CNN	RF	CNN
p01	573	0.148	0.126	0.927	0.928	540	NA *	NA *	NA *	NA *
p02	1029	0.596	0.579	0.850	0.830	864	0.093	0.097	0.904	0.917
p03	521	0.833	0.839	0.921	0.921	362	0.903	0.92	0.840	0.884
p04	356	0.938	0.935	0.913	0.907	641	0.969	0.997	0.916	0.870

* Impaired side use = 0, so cannot be calculated. Accuracies of random forest and CNN models on in-lab and in-home data are highlighted in gray columns.

**Table 4:** Prediction of at-home paretic hand use from machine learning models trained on in-lab data.

PID	Accuracy		Functional Use			
			Predicted		Absolute Error	
	RF	CNN	RF	CNN	RF	CNN
p01	0.980	0.928	0.020	0.311	0.020	0.311
p02	0.620	0.620	0.420	0.420	0.271	0.271
p03	0.800	0.826	0.950	0.925	0.130	0.105
p04	0.874	0.921	0.924	0.969	0.002	0.047

## Data Availability

The raw data supporting the conclusions of this article will be made available by the authors upon request.

## Abbreviations

The following abbreviations are used in this manuscript:

ADL/IADLs	Activities and Instrumental Activities of Daily Living
ARAT	Action Research Arm Test
CNN	Convolution Neural Networks
FAABOS	Functional Arm Activity Behavioral Observation System
RF	Random Forest
UE	Upper extremity
UEFM	Upper-Extremity Fugl–Meyer
